# The association between AI literacy and creative self-efficacy: a moderated mediation model of learning adaptability and teacher support

**DOI:** 10.3389/fpsyg.2026.1798750

**Published:** 2026-07-28

**Authors:** Lu Pan, Xuezhen Shuang, Yiping Liu

**Affiliations:** Department of Water Resources Engineering, Sichuan Water Conservancy Vocational College, Chengdu, China

**Keywords:** AI literacy, artificial intelligence in education, creative self-efficacy, learning adaptability, teacher support

## Abstract

**Background:**

As artificial intelligence (AI) becomes increasingly integrated into education, fostering student creativity is a critical priority. While prior research suggests a positive link between AI literacy and creative self-efficacy, the underlying psychological mechanisms and key contextual factors remain largely unexplored. This study aimed to address this gap by examining the mediating roles of learning adaptability (cognitive, behavioral, and emotional) and the moderating role of teacher support in this relationship.

**Methods:**

A total of 509 university engineering students (68.6% male; *M* age = 19.36 years) participated in a survey. They completed validated measures of AI literacy, learning adaptability, teacher support, and creative self-efficacy. A moderated mediation model was tested using statistical analysis.

**Results:**

The findings indicated a significant positive association between AI literacy and creative self-efficacy. This relationship was significantly mediated by behavioral and emotional adaptability, but not by cognitive adaptability. Furthermore, teacher support moderated the positive effect of AI literacy on emotional adaptability; this effect was stronger for students who perceived higher levels of teacher support.

**Conclusion:**

The study advances a nuanced model demonstrating that AI literacy enhances creative confidence primarily by fostering a willingness to act (behavioral adaptability) and bolstering emotional resilience (emotional adaptability). Teacher support is identified as a crucial contextual resource that amplifies this positive process. These findings offer valuable insights for educators and policymakers aiming to design interventions that maximize the creative potential of AI in learning environments.

## 1 Introduction

The emergence of generative artificial intelligence(AI) has fundamentally transformed the ways individuals learn and live and is increasingly being applied in professional domains such as education (Chen L. et al., 2020; Chen X. et al., 2020; Hwang et al., 2020). Within the educational sphere, AI is conceptualized not merely as a tool but as a constitutive element of the learning process. This paradigm shift reconfigures learning demands by requiring students to interact with, evaluate, and strategically leverage AI-generated information. According to Bandura’ s Social Cognitive Theory (SCT), student learning outcomes are not generated in isolation but are determined by the dynamic interplay of personal factors, behavior, and the environment (Bandura, 2001). As AI technologies become increasingly embedded in classroom instruction, understanding how person–behavior–environment processes jointly shape students’ key psychological competencies has emerged as a critical theoretical and practical priority.

However, a gap exists between the rapid integration of AI into educational practice and students’ preparedness. Specifically, many students demonstrate substantial difficulties in AI-supported learning contexts. For instance, they may overestimate the authority of AI-generated information while overlooking potential inaccuracies (Ng et al., 2021), or they may struggle to formulate precise prompts to obtain high-quality information and fail to effectively identify and rectify AI’ s errors (Kasneci et al., 2023). This capability deficit leads to two conflicting attitudes: an over-reliance on AI outputs, which are accepted uncritically (Baidoo-Anu and Owusu, 2023), and conversely, a sense of mistrust stemming from an inability to accurately calibrate trust (Lee and See, 2004). These challenges indicate that students often struggle to effectively plan monitor, and evaluate learning processes when assisted by AI (Chan and Hu, 2023).

These gaps demonstrate that merely providing access to AI tools is insufficient. Consequently, understanding how AI literacy influences key learning outcomes (such as creative self-efficacy) through the mediation of learning process variables has become an important and urgent research agenda. More importantly, the existing literature has seldom explored whether AI literacy exerts its influence through core psychological processes such as learning adaptability, which encompasses cognitive, behavioral, and emotional dimensions, nor has it adequately considered the role of teacher support as a critical learning resource in AI-enhanced educational settings. Therefore, drawing upon the framework of Social Cognitive Theory, this study constructs an integrative process model to examine whether AI literacy promotes creative self-efficacy, and whether this relationship is mediated by learning adaptability and moderated by teacher support.

### 1.1 AI literacy

Artificial intelligence (AI) technologies are being extensively integrated into the educational domain, fundamentally transforming the learning experience (Zhai et al., 2021). These technologies enable students to customize and personalize course content based on their individual needs, thereby enhancing their learning efficiency and quality (Chen L. et al., 2020; Chen X. et al., 2020). Concurrently, the concept of “AI literacy” has garnered considerable attention. AI literacy is defined as an individual’s proficiency in utilizing AI technologies (Wang et al., 2023). According to Social Cognitive Theory, individuals must develop corresponding personal factors to effectively adapt to environmental changes and engage in successful learning behaviors (Bandura, 1986). In the contemporary context, AI literacy emerges as this pivotal personal factor. It encompasses a range of capabilities, including the ability to critically evaluate AI technologies, communicate and collaborate effectively with them, and leverage them as empowering tools, while also emphasizing an understanding of their ethical and societal implications (Long and Magerko, 2020). AI literacy is broadly viewed as a critical prerequisite for students to navigate future opportunities and challenges, and for the future workforce to collaborate effectively with AI (Cetindamar et al., 2024; Dai et al., 2020). To this end, the global education community has been actively exploring systematic pathways for its cultivation across multiple dimensions, including curriculum development, pedagogical research, and literacy assessment (Laupichler et al., 2022; Touretzky et al., 2019).

### 1.2 AI literacy and creative self-efficacy

Within the personal factors of Social Cognitive Theory, self-efficacy—defined as an individual’s belief in their capability to successfully execute specific behaviors—is a core driver of action (Bandura, 1977). As a specific manifestation of self-efficacy in the domain of creativity, creative self-efficacy directly predicts an individual’s creative intentions and performance (Tierney and Farmer, 2002). It is conceptualized based on theories of self-efficacy and creativity and is defined as the belief that one can produce creative outcomes (Tierney and Farmer, 2002). Serving as a precursor to creativity (Bandura, 1977), empirical evidence has demonstrated that creative self-efficacy is instrumental in fostering creative behaviors and enhancing creative performance (Yeh et al., 2012; Choi, 2004). Consequently, promoting creative self-efficacy has become a critical agenda in education.

Creative self-efficacy is a malleable construct (Tierney and Farmer, 2011), and Bandura’s self-efficacy theory posits that “mastery experiences” are the most effective means of building it. In the current era of artificial intelligence, AI literacy represents the key personal capability that facilitates such mastery experiences. Prior research has suggested that learners with higher AI literacy are likely more adept at using AI tools and technologies, enabling them to interact effectively with AI to generate novel ideas or unexpected perspectives. This accumulation of successful experiences can bolster learners’ confidence in problem-solving, thereby enhancing their creative self-efficacy (Kim et al., 2025). However, despite this clear theoretical link, empirical research specifically examining how AI literacy influences creative self-efficacy remains relatively scarce. More importantly, the underlying psychological mechanisms—such as how learning adaptability functions as a mediator in AI-empowered environments—have been largely unexplored. Therefore, this study aims to address this gap by constructing an integrative model to investigate the impact of AI literacy on creative self-efficacy and its underlying mechanisms.

Based on the literature reviewed, we posit that AI literacy may positively influence students’ creative self-efficacy.

### 1.3 The mediating mechanism: the three dimensions of learning adaptability

Adaptability is defined as “the ability to make an appropriate response to changed or changing circumstances; the ability to modify or adjust one’s behavior in meeting different circumstances or different people” (VandenBos, 2007). Research indicates that young people can purposefully manage novel and evolving academic and non-academic tasks by constructively regulating their personal resources—cognitive, emotional, and behavioral—to achieve positive adjustment (Benight and Bandura, 2004; Martin and Marsh, 2009; Fredricks et al., 2004). As a relevant underlying psychological construct, adaptability can help students function effectively within learning environments (Martin et al., 2012; Martin et al., 2013). In this study, we operationalize learning adaptability into three interconnected dimensions: cognitive, behavioral, and emotional adaptability. Cognitive adaptability refers to an individual’s capacity to flexibly adjust their thinking patterns and cognitive strategies when faced with new knowledge or problems. This includes accurately appraising a situation (cognitive appraisal) and moving beyond fixed cognitive sets to cope with uncertainty (cognitive regulation) (Folkman et al., 1986). Behavioral adaptability pertains to an individual’s ability to proactively adjust or attempt new behaviors in response to task demands and environmental changes during the learning process. This capability implies that an individual can alter the manner and extent of their practices to meet new requirements (Heckhausen et al., 2010; Schulz and Heckhausen, 1996). Emotional adaptability involves an individual’s ability to effectively manage and regulate their emotional states when confronting challenges, uncertainty, and setbacks in learning. The core of this dimension is not the raw emotional impulse but the process of emotion regulation itself, which determines whether an individual can maintain a positive mindset under pressure (Ekman, 1992; Gross and John, 2003).

Learning adaptability serves as a crucial pathway for building creative self-efficacy, primarily by influencing its foundational sources as outlined in Social Cognitive Theory (Bandura, 1997). Specifically: cognitive adaptability builds creative self-efficacy by creating internal, cognitive-level mastery experiences. An individual’s cognitive adaptability is reflected in their cognitive flexibility (Spiro et al., 1991)—the ability to move beyond established mindsets and seek solutions from multiple perspectives when old methods fail (Dietrich, 2004). These successful experiences demonstrate to the individual their capacity for creative thought, reinforcing the belief that “I can solve creative problems.” Behavioral adaptability reinforces creative self-efficacy through external, practice-level success. It manifests as active exploratory behaviors (March, 1991), such as proactively trying new tools, engaging in rapid prototyping (Schrage, 2000), and learning effectively from trial and error (Brown, 2009). These successful practical experiences provide direct evidence that strengthens and enhances creative self-efficacy. Emotional adaptability sustains creative self-efficacy by regulating an individual’s “Physiological and Affective States.” The uncertainty inherent in the creative process can easily trigger negative emotions. However, individuals with high emotional adaptability can reframe setbacks as learning opportunities (Dweck, 2006), thus averting the self-doubt prompted by negative emotional arousal. This positive emotional state protects an individual’s intrinsic motivation and efficacy beliefs in the face of challenges (Amabile, 1996), enabling them to remain engaged in creative activities.

In the current AI-empowered educational context, AI literacy is a key antecedent influencing an individual’s learning adaptability. It provides the necessary knowledge base and skill support for students’ adaptive regulation at the cognitive, behavioral, and emotional levels. Existing research suggests that students with high AI literacy can more accurately construct mental models of how AI works, enabling them to clearly understand its capabilities and limitations (cognitive adaptability) (Long and Magerko, 2020). Concurrently, this preparation in knowledge and skills significantly enhances individuals’ willingness to use AI tools, encouraging them to engage in bolder experimentation and exploration (behavioral adaptability). More importantly, a sense of familiarity and control over the technology can effectively reduce the anxiety and uncertainty individuals experience when confronting new technologies (emotional adaptability) (Venkatesh, 2000).

In summary, AI literacy itself does not directly translate into creative confidence; rather, its value is realized by enhancing students’ learning adaptability. Students equipped with AI literacy are more likely to exhibit cognitive, behavioral, and emotional flexibility and resilience in their learning. It is the series of successful experiences and positive emotional states derived from these adaptive behaviors that shape and elevate an individual’s creative self-efficacy. Therefore, learning adaptability serves as a crucial bridge in this process. We posit that the three dimensions of learning adaptability represent the key psychological mechanisms through which AI literacy influences creative self-efficacy, acting as mediators in this relationship.

### 1.4 The moderating role of teacher support

As a key form of social support in the educational context, teacher support is defined as students’ perception of their teachers’ care, trust, and assistance (Trickett and Moos, 1973; Ryan and Patrick, 2001). According to Self-Determination Theory (SDT), teacher support can be framed as an external environmental factor that satisfies students’ three basic psychological needs: competence, autonomy, and relatedness. The fulfillment of these needs is central to promoting individuals’ intrinsic motivation and positive development (Ryan and Deci, 2000). Building on this framework and integrating prior research in the Chinese educational context (Chen and Guo, 2016; Qiao et al., 2013), this study operationalizes teacher support into three dimensions: competence support, autonomy support, and emotional support. Competence support refers to students’ perception that teachers help them enhance their capabilities to meet challenges and complete tasks, directly satisfying their need for competence (Federici and Skaalvik, 2014). Autonomy support involves teachers understanding and adopting students’ internal perspectives, enabling students to feel that their learning behaviors are self-directed rather than externally coerced, thus fulfilling their need for autonomy (Strati et al., 2017). Emotional support pertains to teachers creating a safe and warm psychological atmosphere by expressing genuine care, empathy, and respect. This form of support helps students feel accepted and connected, thereby satisfying their need for relatedness (Federici and Skaalvik, 2014).

Skinner and Belmont (1993) posited that teacher support is a key antecedent of students’ learning adaptability. Consistent with this theoretical perspective, a substantial body of research has confirmed that teacher support is indeed a critical factor in promoting students’ learning adaptability (Li et al., 2023; Skinner and Belmont, 1993). Specifically, under conditions of high teacher support, students can more effectively translate their AI literacy into cognitive, behavioral, and emotional adaptability. This moderating effect is realized through three primary pathways: First, teacher support facilitates the conversion of AI knowledge into cognitive adaptability by providing “cognitive scaffolding” (Wood et al., 1976). When facing the uncertainty and complexity of AI, mere knowledge is insufficient. Highly supportive teachers guide students on how to critically verify information and teach effective prompting strategies (Baidoo-Anu and Owusu, 2023). This guidance essentially helps students learn how to flexibly adjust their thinking and construct new frames of understanding when encountering cognitive obstacles, thereby fostering their cognitive adaptability. Second, teacher support promotes the translation of AI skills into behavioral adaptability by fostering a proper mindset of “human-AI collaboration.” The powerful capabilities of AI may lead to over-reliance, diminishing students’ motivation for active exploration. Supportive teachers encourage students to position AI as a “creative partner” while guiding them to lead the entire learning process. This guidance effectively prevents the formation of passive dependency, encouraging proactive experimentation and trial-and-error, thus enabling students to apply their AI skills in practice and develop stronger behavioral adaptability. Third, teachers foster emotional adaptability by helping students maintain a positive mindset while using AI. Students inevitably encounter negative emotions such as frustration and anxiety when using AI. Highly supportive teachers can mitigate these emotional impacts through empathetic and caring communication. This emotional backing provides a safe exploratory space for students, empowering them to face challenges and view setbacks as part of the learning process.

In summary, teacher support acts as a “catalyst” that determines how effectively AI literacy is transformed into learning adaptability. When teachers provide sufficient support, students’ ability to adapt to the AI environment at cognitive, behavioral, and emotional levels is significantly enhanced. Therefore, we investigate the potential moderating role of teacher support in both the direct link between AI literacy and creative self-efficacy and the indirect link mediated by the three dimensions of learning adaptability.

### 1.5 The present study

Despite the undeniable importance of AI literacy, the existing literature exhibits several notable limitations. First, research has predominantly focused on the direct association between AI literacy and learning outcomes, often overlooking the underlying psychological mechanisms through which this relationship unfolds. That is, internal cognitive, motivational, and self-regulatory processes remains largely unclear. Second, the mediating role of learning adaptability, as a core psychological resource for coping with and adjusting to emerging technological environments, has received scant scholarly attention in the context of AI-empowered learning. Finally, existing models have frequently overlooked the critical environmental factor of teacher support as a salient form of social and instructional support within AI-enhanced classrooms.

**Figure 1:**
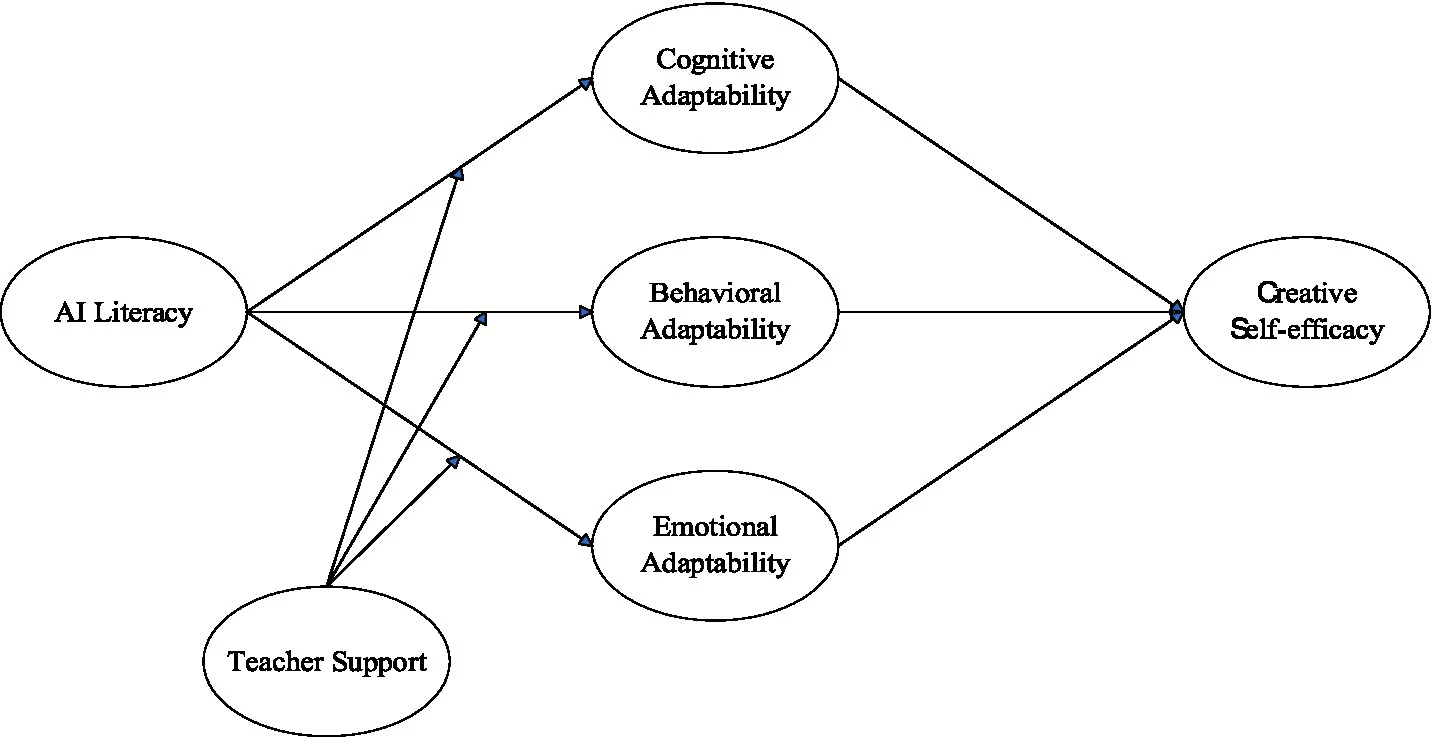
Proposed moderated dual mediation model.

Therefore, drawing upon Bandura’s Social Cognitive Theory and integrating insights from Self-Determination Theory on teacher support, the present study constructs a moderated mediation model, as depicted in Figure 1. The objective is to investigate the mechanisms through which AI literacy influences the creative self-efficacy of university engineering students. Specifically, this study has two primary objectives: (1) to examine the mediating role of the three dimensions of learning adaptability (cognitive, behavioral, and emotional) in the relationship between AI literacy and creative self-efficacy; and (2) by incorporating teacher support as a moderator, to test whether it enables AI literacy to be more effectively translated into student learning adaptability, thereby influencing their creative self-efficacy. The main hypotheses of this study are as follows:

*H1*. AI literacy positively predicts creative self-efficacy.

*H2*. Cognitive adaptability mediates the relationship between AI literacy and creative self-efficacy.

*H3*. Behavioral adaptability mediates the relationship between AI literacy and creative self-efficacy.

*H4*. Emotional adaptability mediates the relationship between AI literacy and creative self-efficacy.

*H5*. Teacher support moderates the relationship between AI literacy and cognitive adaptability, such that the relationship is stronger when teacher support is high.

*H6*. Teacher support moderates the relationship between AI literacy and behavioral adaptability, such that the relationship is stronger when teacher support is high.

*H7*. Teacher support moderates the relationship between AI literacy and emotional adaptability, such that the relationship is stronger when teacher support is high.

## 2 Method

### 2.1 Participants and procedures

Participants were recruited through convenience sampling from a vocational college in Chengdu, Sichuan Province. The sample consisted of first- and second-year undergraduate students majoring in engineering. Data were collected between September and November 2025. Prior to participation, all individuals were provided with detailed information about the study and informed of their right to withdraw at any time. After obtaining informed consent, participants independently completed an online questionnaire on their smartphones via Wenjuanxing, an online survey platform. All participants received course credit as compensation for their participation. The study was approved by the Institutional Review Board (IRB) of the first author’s institution.

A total of 509 participants were initially recruited for the study (68.6% male). Following data screening, responses were excluded if the completion time was less than 180 s or if ten or more consecutive identical answers (i.e., straight-lining) were detected. After applying these exclusion criteria, 485 valid responses were retained for the final analysis, yielding an effective response rate of 95.28%.

### 2.2 Measures

#### 2.2.1 AI literacy

AI literacy was measured using the Chinese version of the AI Literacy Scale for Chinese College Students (AILS-CCS) (Ma and Chen, 2024). This 15-item scale assesses university students’ AI literacy (e.g., “I can use AI applications or products to assist my learning.”). All items were rated on a 5-point Likert scale, (from “1 = *Strongly Disagree*” to “5 = *Strongly Agree*”). Cronbach’s alpha was 0.892.

#### 2.2.2 Teacher support

We measured teacher support with the Chinese version of the Student’s Perceived Teacher Support Scale (Yu et al., 2017). This scale has 15 items measuring students’ perceived teacher support (e.g., “When I encounter difficulties in my studies, I can always find a teacher to help me”), rated on a 5-point scale (from “1 *= Strongly disagree*” to “5 *= Strongly agree*”). Cronbach’s alpha was 0.812.

#### 2.2.3 Learning adaptability

Learning adaptability was measured using the Chinese version of the 9-item Adaptability Scale (Martin et al., 2012). The scale is composed of three subscales: Cognitive Adaptability (3 items; e.g., “I am able to think through a number of possible options to help me deal with a new situation”), Behavioral Adaptability (3 items; e.g., “I am able to change my behavior to suit different situations”), and Emotional Adaptability (3 items; e.g., “I am able to deal with pressure so it does not get in the way of what I have to do”).

All items were rated on a 7-point Likert scale, ranging from 1 (*Strongly disagree*) to 7 (*Strongly agree*). A higher average score indicates greater learning adaptability. The Cronbach’s alpha coefficients were 0.930 for cognitive adaptability, 0.927 for behavioral adaptability, and 0.915 for emotional adaptability.

#### 2.2.4 Creative self-efficacy

We measured creative self-efficacy with the Chinese version of the Creative Self-Efficacy Questionnaire (Hong and Lin, 2004). This scale has 14 items measuring university students’ creative self-efficacy (e.g., “When facing a challenging task, I am confident that I can recall a lot of relevant knowledge”), rated on a 4-point scale (from “1 = *Not at all true*” to “4 = *Exactly true*”). Cronbach’s alpha was 0.762.

### 2.3 Data analysis

First, Descriptive statistics, Pearson correlation analysis, and internal consistency reliability were calculated for the study variables using the R environment for statistical computing (Version 4.5.1). Second, we tested a mediation model in Mplus 7.4 (Muthén and Muthén) to examine the mediating role of learning adaptability (i.e., cognitive, behavioral, and emotional adaptability) in the relationship between AI literacy and creative self-efficacy. The significance of the mediating effects was assessed using Maximum Likelihood (ML) estimation with bootstrapping (5,000 resamples) and its 95% confidence intervals (CI). Third, building on the mediation model, we introduced teacher support as a moderator to test for a moderated mediation effect. Specifically, we examined whether teacher support moderated the path from AI literacy to learning adaptability. If this interaction effect was significant, we further probed it by analyzing the simple slopes and the conditional indirect effects. Across all models, age and gender were included as covariates. The significance of the moderated mediation effect was determined using the numerical integration method with 5,000 iterations and its 95% confidence intervals (CI). All reported coefficients are standardized.

## 3 Result

### 3.1 Descriptive statistics and bivariate correlation

The descriptive results were summarized in Table 1. As shown in Table 1, AI literacy was positively associated with behavioral adaptability (*r* = 0.437, *p* < 0.001), emotional adaptability (*r* = 0.384, *p* < 0.001), as well as creative self-efficacy (*r* = 0.712, *p* < 0.001). Creative self-efficacy was positively associated with cognitive adaptability (*r* = 0.192, *p* < 0.001), behavioral adaptability (*r* = 0.489, *p* < 0.001), as well as emotional adaptability (*r* = 0.436, *p* < 0.001). Teacher support was positively associated with AI literacy (*r* = 0.157, *p* < 0.01), cognitive adaptability (*r* = 0.119, *p* < 0.001), behavioral adaptability (*r* = 0.132, *p* < 0.01), as well as emotional adaptability (*r* = 0.165, *p* < 0.001).

**Table 1:** The means, standard deviations, reliabilities and correlations among the variables.

Variables	1	2	3	4	5	6
Key variables						
1. AI literacy						
2. Cognitive adaptability	−0.015					
3. Behavioral adaptability	0.437^***^	0.165^***^				
4. Emotional adaptability	0.384^***^	0.034	0.437^***^			
5. Teacher support	0.157^**^	0.119^**^	0.125^**^	0.165^***^		
6. Creative self-efficacy	0.712^***^	0.192^***^	0.489^***^	0.436^***^	0.278^***^	
Covariates						
*M*	3.073	3.783	4.014	3.744	2.961	2.959
*SD*	0.980	1.668	1.681	1.646	0.938	0.868
α	0.892	0.930	0.927	0.915	0.812	0.762

^*^p < 0.05, ^**^p < 0.01, ^***^p < 0.001.

### 3.2 The mediating role of learning adaptability

**Figure 2:**
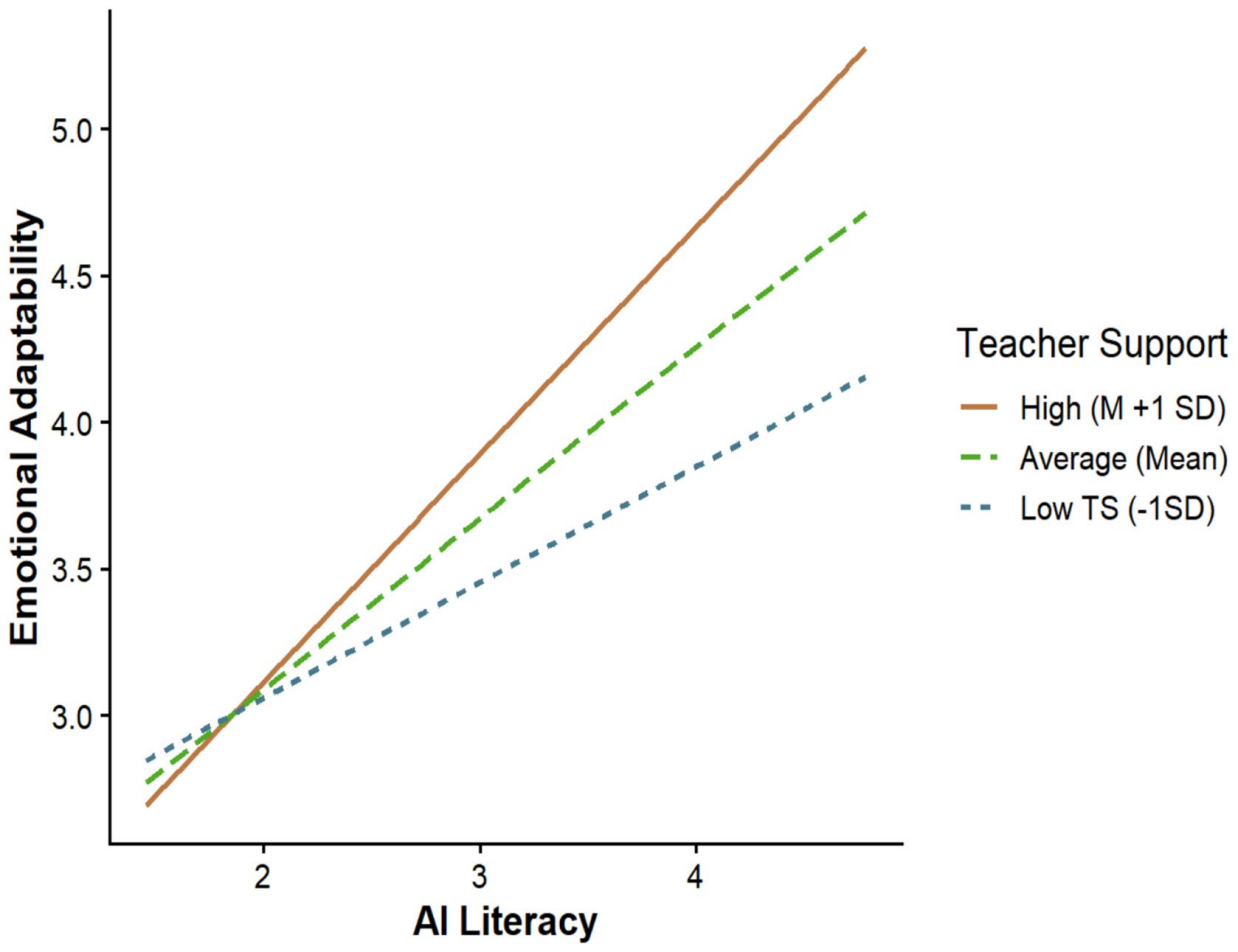
Simple slopes of AI literacy on emotional adaptability at different levels of teacher support.

As depicted in Figure 2 and Table 2, AI literacy was positively related to behavioral adaptability (*β* = 0.478, *SE* = 0.041, *p* < 0.001, 95% CI = [0.391, 0.555]) and emotional adaptability (*β* = 0.429, *SE* = 0.044, *p* < 0.001, 95% CI = [0.339, 0.511]), as well as creative self-efficacy (*β* = 0.737, *SE* = 0.050, *p* < 0.001, 95% CI = [0.488, 0.667]). However, the path from AI literacy to cognitive adaptability (*β* = −0.010, *SE* = 0.052, *p* = 0.852, 95% CI = [−0.027, 0.019]) was not statistically significant, Furthermore, cognitive adaptability (*β* = 0.220, *SE* = 0.035, *p* < 0.001, 95% CI = [0.155, 0.289]), behavioral adaptability (*β* = 0.116, *SE* = 0.045, *p* = 0.010, 95% CI = [0.013, 0.099]), and emotional adaptability (*β* = 0.142, *SE* = 0.045, *p* = 0.002, 95% CI = [0.024, 0.109]) were all positively related to creative self-efficacy.

**Table 2:** Direct, indirect, and total effects of AI literacy on creative self-efficacy.

Indirect effects	*β*	*SE*	*p*	95% CI
Panel A: direct effects				
AI Literacy → cognitive adaptability	−0.010	0.052	0.852	[−0.185,0.154]
AI Literacy → behavioral adaptability	0.478	0.042	*p* < 0.001	[0.391,0.555]
AI Literacy →emotional adaptability	0.429	0.044	*p* < 0.001	[0.339,0.511]
Cognitive Adaptability → creative self-efficacy	0.220	0.035	*p* < 0.001	[0.155,0.289]
Behavioral Adaptability → creative self-efficacy	0.116	0.045	0.010	[0.013,0.099]
Emotional Adaptability → creative self-efficacy	0.142	0.045	0.002	[0.024,0.109]
AI Literacy → creative self-efficacy (c′, direct)	0.737	0.050	*p* < 0.001	[0.488,0.667]
Panel B: indirect effects				
Path 1: AI literacy → cognitive adaptability → creative self-efficacy	−0.002	0.012	0.853	[−0.027,0.019]
Path 2: AI literacy → behavioral adaptability → creative self-efficacy	0.056	0.022	0.010	[0.012, 0.079]
Path 3: AI literacy →emotional adaptability → creative self-efficacy	0.061	0.019	0.001	[0.018, 0.078]
Panel C: total effect				
AI literacy → creative self-efficacy (c, total)	0.851	0.034	*p* < 0.001	[0.052, 0.140]

Results of the mediation analyses showed that the mediation effects of behavioral adaptability (Indirect Effect = 0.056, *SE* = 0.022, *p* = 0.010, 95% CI = [0.012, 0.079]) and emotional adaptability (Indirect Effect = 0.061, *SE* = 0.018, *p* = 0.001, 95% CI = [0.018, 0.078]) were significant. In contrast, the indirect effect through cognitive adaptability was not statistically significant (Indirect Effect = −0.002, *SE* = 0.012, *p* = 0.853, 95% CI = [−0.185, 0.154]), as the confidence interval included zero. This non-significant mediation appears to stem primarily from the non-significant path from AI literacy to cognitive adaptability, despite the significant association between cognitive adaptability and creative self-efficacy.

### 3.3 The moderating role of teacher support

As detailed in Tables 3, 4, the latent moderated mediation model fit the data well. The model without interaction terms showed an excellent fit (χ^2^/d*f* = 1.585, RMSEA = 0.035, CFI = 0.986, TLI = 0.982, SRMR = 0.031). Crucially, a model comparison revealed that incorporating the interaction terms via the Latent Moderated Structural Equations (LMS) method resulted in a significant improvement in model fit (*p* < 0.001), justifying the moderation analysis (Klein and Moosbrugger, 2000; Muthén and Muthén, 2017).

**Table 3:** Model fit indices.

Fit index	Model without interaction	Model with interaction	Recommended criterion
AIC	28455.031	26134.169	Lower is better
H_0_	−14159.515	−12986.085	N/A
d*f*	68	81	N/A
χ^2^/d*f*	1.585	—	< 3.00
RMSEA	0.035	—	<0.06
SRMR	0.031	—	<0.08
CFI	0.986	—	>0.95
TLI	0.982	—	>0.95

**Table 4:** Results of model comparison for the latent moderation effect.

Model comparison	Model without interaction	Model with interaction	Test statistics	Significance
AIC	28455.031	26134.169	2320.862	Significant
H_0_	−14159.515	−12986.085	*p* < 0.001	Significant
No. of parameters	68	81		

**Figure 3:**
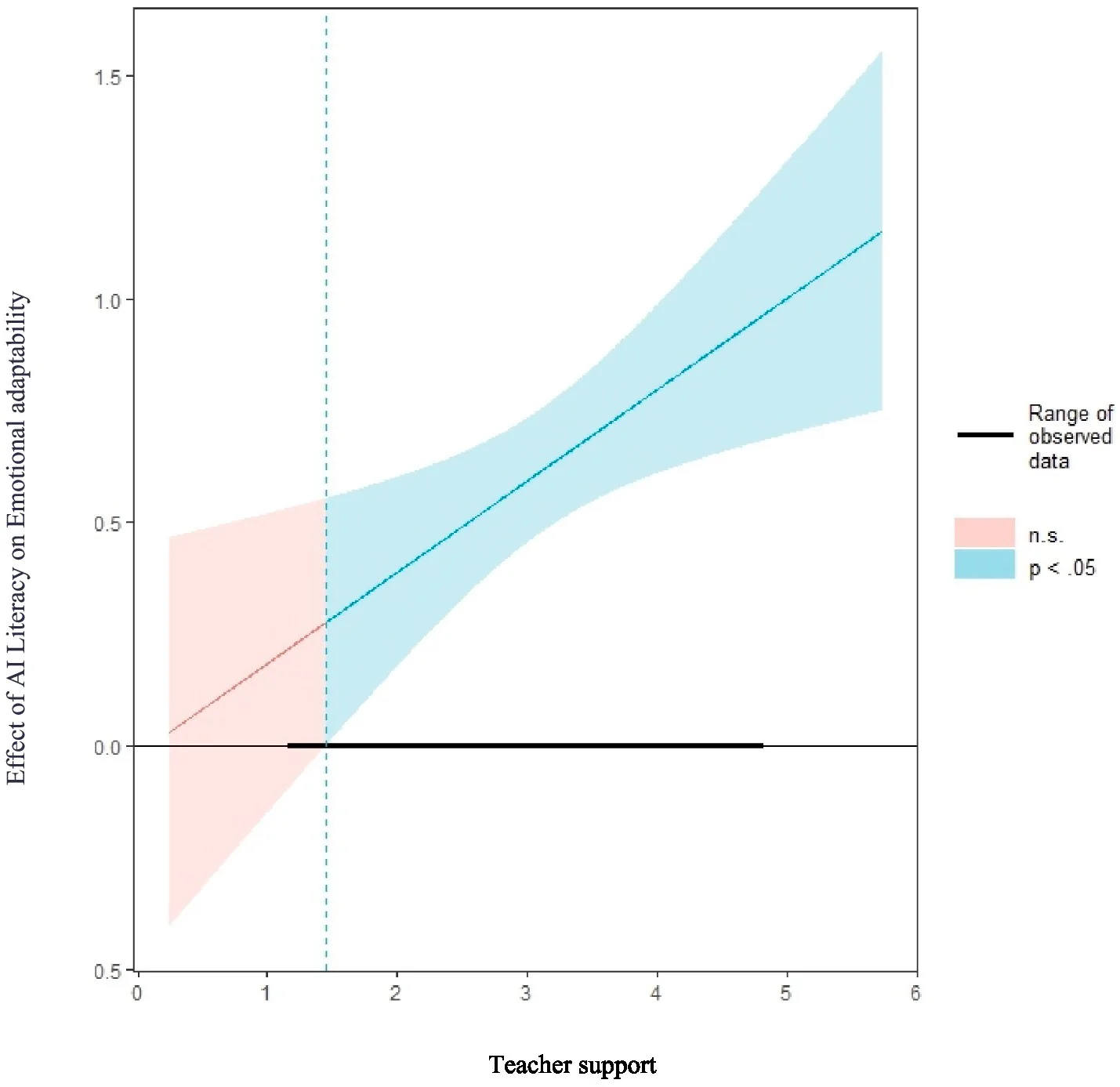
The relation between AI literacy and emotion adaptability moderated by teacher support.

As shown in Table 5, teacher support significantly moderated the relationship between AI literacy and emotional adaptability (*β* = 0.117, *SE* = 0.050, *p* = 0.021). Simple slope analyses (Table 6 and Figure 3) revealed that the effect of AI literacy on emotional adaptability was significant at low (*β* = 0.274, *SE* = 0.083, 95% CI [0.124, 0.492]), average (*β* = 0.391, *SE* = 0.056, 95% CI [0.317, 0.562]), and high (*β* = 0.057, *SE* = 0.066, 95% CI [0.424, 0.717]) levels of teacher support, with the effect strengthening as teacher support increased. Johnson-Neyman analysis further indicated that the effect of AI literacy on emotional adaptability became significant when teacher support exceeded 1.45 (observed range: 1.18–4.79), suggesting that the facilitative role of AI literacy was evident for the vast majority of participants in this sample (see Figure 3). The conditional indirect effects of AI literacy on creative self-efficacy via emotional adaptability were also significant at low (*β* = 0.026, *SE* = 0.010, 95% CI [0.008, 0.048]), average (*β* = 0.038, *SE* = 0.011, 95% CI [0.017, 0.061]), and high (*β* = 0.051, *SE* = 0.015, 95% CI [0.023, 0.081]) levels of teacher support, and the index of moderated mediation was significant (index = 0.013, SE = 0.007, 95% CI [0.003, 0.028]),confirming that the mediated pathway was conditionally dependent on teacher support.

**Table 5:** The moderated mediation model.

Variables	Behavioral adaptability (*R*^2^ = 0.576)			Emotional adaptability (*R*^2^ = 0.682)			Creative self-efficacy (*R*^2^ = 0.527)		
	*β*	*SE*	*p*	*β*	*SE*	*p*	*β*	*SE*	*p*
Key variables									
AI Literacy	0.477	0.041	<0.001	0.391	0.049	<0.001	0.740	0.038	<0.001
Teacher support	0.002	0.048	0.963	0.123	0.052	0.011	0.242	0.091	0.008
AI literacy ×teacher support	0.028	0.045	0.535	0.117	0.050	0.021	—	—	—
Behavioral adaptability	—	—	—	—	—	—	0.113	0.045	0.012
Emotional adaptability	—	—	—	—	—	—	0.145	0.042	0.001

**Table 6:** Simple slopes and conditional indirect effects of AI literacy on emotional adaptability and creative self-efficacy at different levels of teacher support.

Level of teacher support	*β*	*SE*	95%CI
Simple slopes: AI literacy → emotional adaptability			
Low (M − 1 SD)	0.274	0.083	[0.124, 0.492]
Average (Mean)	0.391	0.056	[0.317, 0.562]
High (M + 1 SD)	0.507	0.066	[0.424, 0.717]
Conditional indirect effects: AI literacy → emotional adaptability → creative self-efficacy			
Low (M − 1 SD)	0.026	0.010	[0.008, 0.048]
Average (Mean)	0.038	0.011	[0.017, 0.061]
High (M + 1 SD)	0.051	0.015	[0.023, 0.081]
Index of moderated mediation	0.013	0.007	[0.003, 0.028]

**Figure 4:**
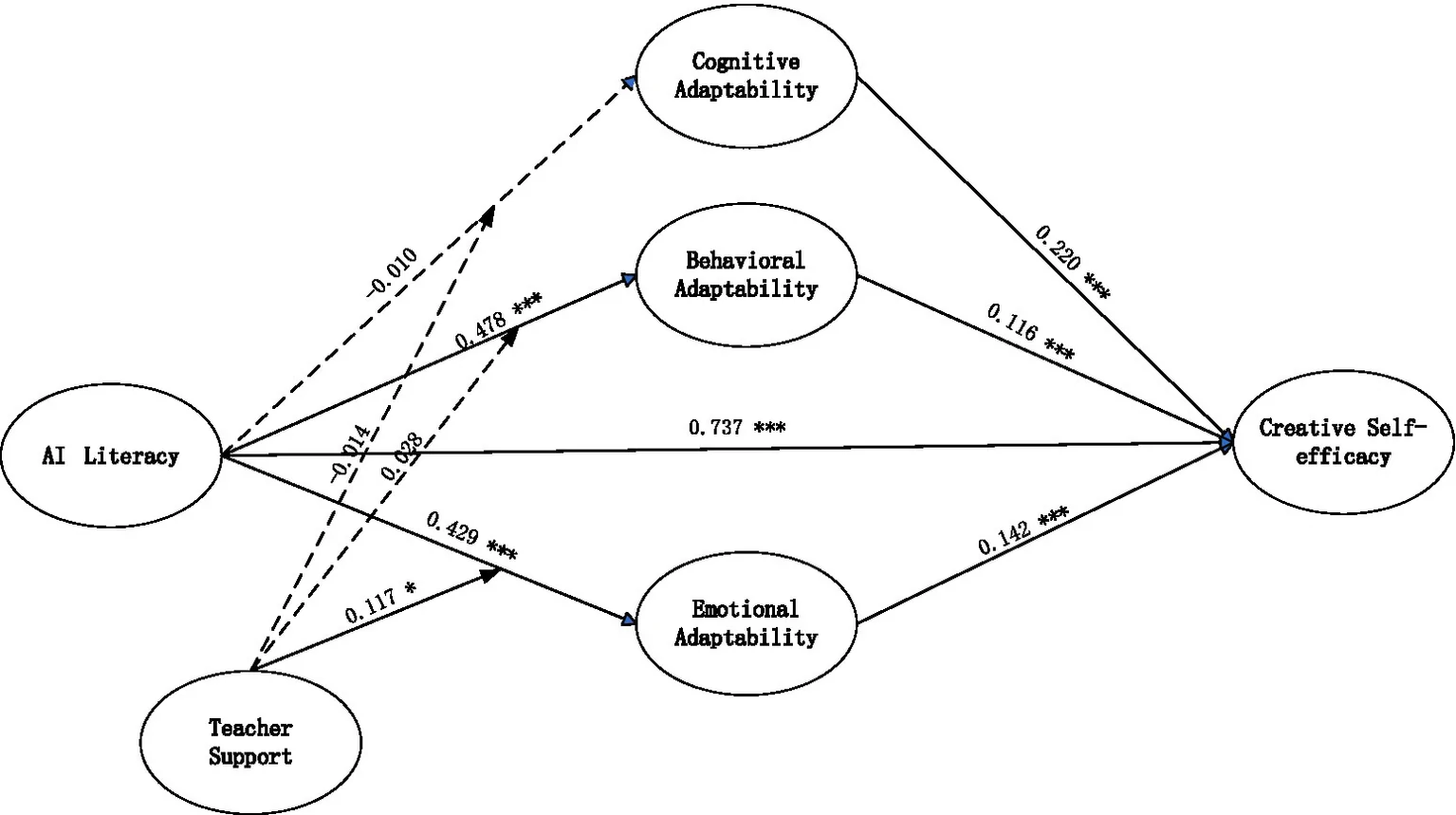
Structural model result. Standardized coefficients are reported. *p < 0.05, **p < 0.01, ***p < 0.001.

To provide a concise overview of the hypothesis testing outcomes, Table 7 summarizes whether each hypothesis was supported or not supported based on the statistical analyses reported above. The final structural model result is presented in Figure 4.

**Table 7:** Hypothesis testing result.

Hypothesis	Path	*β*	*SE*	*p*	95% CI	Result
H_1_	AI literacy → creative self-efficacy	0.737	0.050	*p* < 0.001	[0.488,0.667]	Supported
H_2_	AI literacy → cognitive adaptability →creative self-efficacy	−0.002	0.012	0.853	[−0.027, 0.019]	Not supported
H_3_	AI literacy →behavioral adaptability →creative self-efficacy	0.056	0.022	0.010	[0.012, 0.079]	Supported
H_4_	AI literacy →emotional adaptability →creative self-efficacy	0.061	0.019	0.001	[0.018, 0.078]	Supported
H_5_	AI literacy × teacher support → cognitive adaptability	−0.014	0.063	0.516	[−0.163, 0.082]	Not supported
H_6_	AI literacy × teacher support → behavioral adaptability	0.028	0.045	0.535	[−0.163, 0.117]	Not supported
H_7_	AI literacy × teacher support → emotional adaptability	0.117	0.050	0.021	[0.018, 0.215]	Supported

## 4 Discussion

The present study employed a moderated mediation model to investigate the psychological mechanisms linking AI literacy and creative self-efficacy. The findings revealed that AI literacy was positively associated with creative self-efficacy, a relationship that was mediated by learning adaptability. Within this framework, this study is among the first to offer a comprehensive explanation of how and under what conditions AI literacy translates into creative self-efficacy. The results highlight the pivotal roles of learning adaptability and teacher support in the process through which AI literacy fosters creative self-efficacy.

AI literacy was found to be positively associated with creative self-efficacy, lending further support to Social Cognitive Theory (Bandura, 1986). As a key personal competence rooted in a deep understanding and effective application of AI tools, AI literacy empowers learners to engage in a series of creative practices. Such practices—for instance, using AI to explore diverse perspectives, generate novel ideas, or optimize solutions—transform the learning process into a cycle of continuous positive feedback, which in turn significantly enhances an individual’ s confidence in tackling creative challenges. Therefore, AI literacy constitutes a key personal resource for enhancing students’ creative self-efficacy.

### 4.1 The mediating roles of behavioral and emotional adaptability

Through mediation analysis, the present study found that AI literacy significantly and positively predicted creative self-efficacy, primarily through the pathways of behavioral and emotional adaptability, whereas the mediating role of cognitive adaptability was not supported. Specifically, AI literacy not only enhanced students’ willingness to explore in practice (behavioral adaptability) but also bolstered their psychological resilience in the face of setbacks (emotional adaptability). It thereby enhances individuals’ confidence in producing creative outcomes through the dual channels of ‘action’ and ‘mindset’. These findings are highly consistent with Bandura’s (1997) self-efficacy theory regarding the sources of efficacy beliefs. The theory posits that ‘mastery experiences,’ the most powerful source of efficacy, and ‘physiological and emotional states,’ a key influential factor, are shaped precisely through an individual’s practical actions and emotional experiences. On one hand, individuals with high AI literacy are more willing to proactively try and explore AI tools (behavioral adaptability), and these successful practical experiences directly build and reinforce their belief that “I can create” (Tierney and Farmer, 2011). Importantly, the unique features of AI-enabled learning environments—such as the high uncertainty in human-computer interaction and the rapid, iterative process of trial and error—serve to amplify this effect, making firsthand success the most direct and potent evidence for enhancing creative confidence.

On the other hand, contrary to our initial hypothesis (H2), this study did not find a mediating role for cognitive adaptability, which is an equally important finding worthy of discussion. Compared to behavioral and emotional adaptability, the pathway of cognitive adaptability may have been overestimated in the context of AI literacy research. One possible explanation is that AI literacy itself already encompasses a rich set of cognitive elements (e.g., understanding AI principles, critical thinking). Consequently, once students possess a high level of AI literacy, its primary contribution to creative self-efficacy may no longer be about ‘thinking more flexibly’ (cognitive adaptability), but rather about translating existing cognitive abilities into ‘acting more boldly’ (behavioral adaptability) and ‘maintaining a more stable mindset’ (emotional adaptability). From the perspective of Social Cognitive Theory (Bandura, 2001), although AI literacy is a cognitive ‘personal factor,’ its primary function in fostering creative confidence may be to unlock the ‘behavioral’ and ‘emotional’ mechanisms that generate direct feedback. Merely thinking flexibly in one’s mind, without the courage to put ideas into practice and the resilience to face setbacks, may be insufficient to translate into a firm belief in one’s own creative capabilities.

In summary, the present study identifies a dual-pathway mechanism through which AI literacy influences creative self-efficacy: namely, by enhancing behavioral and emotional adaptability. These findings advance our understanding of the mechanisms for cultivating creative confidence in AI-enabled environments and provide a psychological foundation for future educational interventions. Future research could further explore how to design pedagogical activities that help students more effectively translate AI-related cognitive knowledge into exploratory behaviors and positive emotional experiences, thereby maximizing the positive impact of AI literacy on student development.

### 4.2 The moderating role of teacher support

This study further examined the moderating role of teacher support in the process through which AI literacy translates into learning adaptability. Specifically, teacher support significantly strengthened the positive effect of AI literacy on emotional adaptability: compared to individuals who perceived low teacher support, those in high-support environments were able to more effectively leverage their AI literacy to foster positive emotional adaptability. This finding supports the core tenet of Self-Determination Theory (SDT) concerning external environmental support (Ryan and Deci, 2000), which posits that a supportive environment that satisfies students’ psychological needs is key to promoting their positive development. In a high-support context, the “emotional support” provided by teachers through empathy and care creates a safe exploratory space for students. This encourages them to interpret setbacks with AI as learning opportunities rather than personal failures, thereby protecting and enhancing their emotional resilience. In contrast, in an environment lacking teacher support, difficulties encountered while using AI are more likely to directly trigger anxiety and uncertainty, thus undermining the positive emotional experiences that AI literacy should otherwise facilitate. These findings are consistent with previous research. Research has consistently shown that teachers’ emotional support can effectively buffer students against negative emotions when using technology in learning.

However, a finding that warrants equally careful consideration is that the moderating role of teacher support on the relationship between AI literacy and behavioral adaptability was not supported. This result may shed light on the core drivers of ‘behavioral adaptability’. One possible explanation is that behavioral adaptability is more directly driven by an individual’s AI literacy and intrinsic motivation rather than being modulated by external social support. Once students have truly mastered AI skills and possess high AI literacy, they are already equipped with the “competence capital” to engage in behavioral exploration. The intrinsic drive stemming from this competence may be so potent that the marginal effect of external teacher support becomes non-significant. In other words, the core value of teacher support may lie more in providing the “emotional courage to dare to err” to foster emotional adaptability, rather than in directly influencing behavioral adaptability by enhancing the “technical behaviors of how to err.” Consequently, the additional moderating effect at the behavioral level is not prominent.

In summary, this study finds that teacher support plays a precise, rather than a generalized, moderating role in the conversion of AI literacy into learning adaptability, with its effect primarily concentrated on the emotional adaptability pathway. This finding not only extends the application of Self-Determination Theory to the field of AI education but also reveals an “emotional catalyst” mechanism of teacher support. It does not universally enhance all dimensions of adaptability but rather acts as a critical emotional resource that specifically activates the pathway from AI literacy to positive emotional experiences, thereby serving as a powerful psychological buffer in AI-enabled learning environments.

### 4.3 Limitations and future research

While the present study yields several valuable conclusions, some limitations should be acknowledged that warrant attention in future research. First, this study employed a cross-sectional design, which precludes definitive causal inferences between variables. Although our hypotheses were supported by the data, the temporal precedence among the variables could not be strictly established. Future studies should adopt longitudinal or experimental designs to more rigorously test the moderated mediation model proposed herein. Second, all data were collected through self-report questionnaires, which may be susceptible to social desirability effects or common method bias. For instance, students might have overestimated their AI literacy or creative self-efficacy. Future research should integrate multi-source, multi-method data, such as behavioral observations of students’ AI usage processes or by introducing objective, task-based assessments of AI literacy. This would mitigate the bias of a single method and provide a more accurate depiction of the learning process in AI-enabled environments. Third, the sample of this study consisted solely of university engineering students, which may limit the generalizability of the findings. Engineering students may possess specific characteristics regarding their logical thinking and technology acceptance. Expanding the research sample to include students from diverse academic backgrounds, such as the humanities and arts, as well as from different educational levels and cultural contexts, would be beneficial for testing the cross-contextual stability of the mechanisms identified in this study. Fourth, the present study measured AI literacy as a holistic construct. However, according to the existing literature (Long and Magerko, 2020), AI literacy is a multidimensional competency framework comprising different facets such as usage skills, critical thinking, and ethical understanding. It is plausible that these different dimensions of literacy have differential effects on learning adaptability. Future research could delve deeper into how each sub-dimension of AI literacy functions through distinct adaptability pathways, thereby providing more fine-grained guidance for the cultivation of AI literacy.

### 4.4 Practical implications

This study offers significant practical implications for educational innovation and the cultivation of student creativity in the age of artificial intelligence.

First, the findings underscore the urgent necessity of shifting AI literacy education from superficial knowledge dissemination to deep competency integration. Although many universities have introduced AI-related courses, they often remain at the level of theoretical introductions or basic tool operations, failing to effectively foster students’ creative self-efficacy. Our research demonstrates that true AI literacy is not an isolated technical skill but a comprehensive competence that drives creativity in practice. Therefore, the focus of higher education should shift from merely “offering AI courses” to “deeply integrating AI into disciplinary learning.” In practical terms, educators could embed AI-based projects into core engineering courses, organize interdisciplinary innovation competitions with industry mentors, and establish co-creation studios for cross-disciplinary problem-solving. Such approaches help students internalize AI literacy through authentic practice, laying a foundation for creative development and career adaptability.

Second, the dual mediating pathways of behavioral and emotional adaptability suggest that interventions should simultaneously target students’ “hands-on” capabilities and their psychological resilience. Educators should move beyond theoretical instruction and design “low-risk, high-exploration” practical tasks to stimulate behavioral adaptability, enabling students to accumulate “I can do it” mastery experiences through action. Concurrently, the study highlights the critical role of emotional adaptability, indicating that educators must also focus on cultivating students’ ability to regulate their emotions when faced with technological uncertainty. To strengthen emotional adaptability, strategies may include reflective journaling on AI-related struggles, peer-sharing of failures and recovery experiences, and mindfulness modules within AI courses. These practices help students reframe technological setbacks as growth opportunities rather than threats to competence.

Third, the precise moderating effect of teacher support on the emotional adaptability pathway emphasizes the importance of building supportive teacher-student relationships during the transition to AI-integrated education. Teacher support is not a vague form of caring but functions as a critical “emotional buffer.” This finding suggests that professional development for teachers is paramount. Such training should not only focus on the technology itself but also equip teachers with the skills to identify and respond to students’ emotional needs during human-computer interaction, create a climate of psychological safety, and guide students to reframe setbacks as growth opportunities. University administrators should provide teachers with the necessary resources and support to enable them to offer students a psychological cushion against the frustrations of AI exploration. Institutional support may include faculty training in emotionally responsive AI pedagogy, mentorship programs for students showing AI-related anxiety or disengagement, and AI learning clinics offering both technical and emotional guidance. This, in turn, will effectively promote the development of creative self-efficacy and maximize the positive impact of AI technology in education.

Beyond the classroom, corporate training in AI-driven industries (e.g., technology, engineering, healthcare) can foster innovation by combining hands-on experimentation with mentorship-based emotional support. Policymakers may likewise embed behavioral and emotional competencies into AI literacy curricula while investing in teacher well-being. Together, these multi-level strategies can maximize the positive impact of AI across educational and professional settings.

## 5 Conclusion

This study developed and empirically tested a moderated mediation model to elucidate the process through which AI literacy enhances students’ creative self-efficacy. By integrating the core dimensions of learning adaptability and the key contextual factor of teacher support, the findings provide a more nuanced understanding of how creative confidence is cultivated in AI-enabled learning environments. This research yielded three major conclusions. This research yielded three key conclusions. First, AI literacy serves as a robust positive predictor of students’ creative self-efficacy, highlighting the foundational role of mastering AI-related competencies in cultivating individuals’ innovative confidence. Second, this study uncovered the dual mediating pathways through which AI literacy influences creative self-efficacy: behavioral and emotional adaptability played key mediating roles, whereas the mediating effect of cognitive adaptability was not significant. This finding suggests that translating AI knowledge into the exploratory behavior of ‘daring to act’ and the positive, resilient mindset of being ‘fearless of failure’ constitutes the core psychological mechanism for enhancing creative confidence. Third, teacher support does not universally enhance all adaptability dimensions; its moderating effect is primarily concentrated on the positive relationship between AI literacy and emotional adaptability, revealing that teacher support serves as a critical ‘emotional buffer’ in AI education. Taken together, these findings offer a novel theoretical perspective on how AI literacy is translated into individual creative confidence. Furthermore, they provide an empirical foundation for the design of more effective AI literacy curricula and teacher support strategies in higher education, ultimately contributing to the cultivation of students’ innovative capabilities in the era of intelligent technologies.

## Data Availability

The original contributions presented in the study are included in the article/supplementary material, further inquiries can be directed to the corresponding author.
