# Correction: The association between AI literacy and creative self-efficacy: a moderated mediation model of learning adaptability and teacher support

**DOI:** 10.3389/fpsyg.2026.1980102

**Published:** 2026-10-14

**Authors:** Lu Pan, Xuezhen Shuang, Yiping Liu

**Affiliations:** Department of Water Resources Engineering, Sichuan Water Conservancy Vocational College, Chengdu, China

**Keywords:** AI literacy, artificial intelligence in education, creative self-efficacy, learning adaptability, teacher support

An incorrect in-text citation was included in section **4 Discussion**, paragraph 2. Yu et al. (2017) concerns teacher support, learning motivation, and academic achievement and does not directly address the relationship between AI literacy and creative self-efficacy. The citation has therefore been removed from this sentence.

A correction has been made to section **4 Discussion**, paragraph 2:

“AI literacy was found to be positively associated with creative self-efficacy, lending further support to Social Cognitive Theory (Bandura, 1986).”

The reference for Li et al. (2023) was erroneously written as Collie, R. J., Martin, A. J., and Bobis, J. (2017). A model of teachers' motivational approach: the role of teachers' achievement goals, perceived classroom demands, and collegial support. *Teach. Teach. Educ*. 68, 21–29.

It should be: Li, J., Huang, J.-H., and Chen, J.-Y. (2023). Relationship between hardiness and the learning adaptation of Chinese college students: the mediating role of teacher support and the moderating role of an only-child status. *Asia Pac. Educ. Res*. 32, 753–768. doi: 10.1007/s40299-022-00692-z

An inaccurate in-text citation was included in Section 1.4 “*The moderating role of teacher support*,” paragraph 2. The citation Collie et al. (2017) has been replaced with Li et al. (2023), which provides direct empirical support for the positive role of teacher support in students' learning adaptation.

A correction has been made to section 1.4 “*The moderating role of teacher support*,” paragraph 2: “Skinner and Belmont (1993) posited that teacher support is a key antecedent of students' learning adaptability. Consistent with this theoretical perspective, a substantial body of research has confirmed that teacher support is indeed a critical factor in promoting students' learning adaptability (Li et al., 2023; Skinner and Belmont, 1993).”

These corrections do not affect the data, analyses, results, interpretation, or conclusions of the article.

The original version of this article has been updated.

